# Differentiating Gender and Sex in Dental Research: A Narrative Review

**DOI:** 10.1155/2022/2457748

**Published:** 2022-08-23

**Authors:** Alice Alberti, Benedetta Morandi, Luca Francetti, Silvio Taschieri, Stefano Corbella

**Affiliations:** ^1^Department of Biomedical Surgical and Dental Sciences, Università Degli Studi di Milano, Milan, Italy; ^2^IRCCS Istituto Ortopedico Galeazzi, Milan, Italy; ^3^Department of Oral Surgery, Institute of Dentistry, I. M. Sechenov First Moscow State Medical University, Moscow, Russia

## Abstract

While in humans the term “sex” refers to the biological attributes that distinguish subjects as male, female, and intersex, the term “gender” refers to psychological, social, and cultural factors that strongly influence attitudes, behaviors, and relationships of individuals. Recently, it has been emphasized how the integration of these two terms in the design of the research can improve the methodology of the research itself. However, in dental research, the influence of gender has not gained enough consideration and it is often used indiscriminately as a synonym for sex. This narrative review discusses the usefulness of considering gender and sex in dental research, whose guidelines have been provided so far on this topic, and whether the top 20 dental scientific journals promote the analysis of sex and gender in their guidelines. Sex and gender analysis in dental research could be important both for analyzing biological differences such as those in the immune or neuro-immune system, cardiovascular physiology, developmental anomalies or deformities, and psychosocial differences such as lifestyle, pain experience and prevalence of chronic pain, eating behavior, and access to healthcare services. As for the specific policies for sex and gender analysis and reporting, only five out of 20 biomedical journals have included them in their editorial policy, which refers mainly to the correct use of the terms “sex” and “gender.” In conclusion, we found that no specific and differentiated sex and gender analysis and reporting are required in dental journals. Their integration, which is still not routinely applied, may be improved in the future by updating editorial guidelines and developing more specific methodological recommendations.

## 1. Introduction

In humans, sex refers to the biological attributes that distinguish subjects as male, female, and intersex [1]. Sexual characteristics are not limited to the reproductive apparatus but include appearance, physiology, behavior, and neuroendocrine and metabolic systems. Sex can be defined through the analysis of various factors: sex-determining genes (XX/XY for most mammals); gametes, i.e., the morphologically distinct type of germ cells that males and females produce; and morphological features including primary (reproductive organs) and secondary sex characteristics (phenotypic traits which become evident at puberty upon hormonal stimulation, such as breast development and wider pelvis in females, and greater muscle mass and more facial and body hair in males).

**Figure 1:**
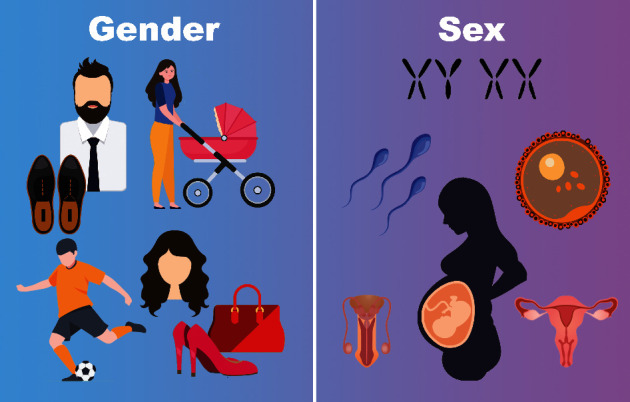
Some examples that underline the difference between sex and gender. Sex is associated with biological attributes, such as sex-determining genes, the reproductive apparatus, morphology of gametes (sperm, ovum), and reproductive physiology. Gender refers to socio-cultural factors, such as activities, professions, or roles associated with a particular gender in a certain society or social context, colors, clothing, and hairstyles.

On the other hand, gender refers to psychological, social, and cultural factors that strongly influence the attitudes, behaviors, and relations of individuals [1]. It is a multi-dimensional concept that includes gender norms, gender identity, and gender relations. Gender norms, which reinforce gender stereotypes, are social and cultural attitudes and expectations about which behaviors, preferences, or professions are appropriate for a certain gender. They are constantly modified and they may vary in specific social contexts. Gender identity refers to how individuals perceive and present themselves, while gender relations are the social interactions that are based on sex, gender identity, and gender norms.  Figure 1 illustrates the difference between sex and gender through some examples.

In humans, neither sex nor gender is binary variables. As for sex, we can distinguish between male, female, and intersex people. As for gender, men and women can be referred to as cisgender or transgender depending on the correspondence or not with the birth-assigned sex; the term nonbinary defines gender-nonconforming individuals which are those whose identity cannot be defined as man or woman and includes several nuances of gender identities between, outside or beyond the gender binary (e.g., gender fluid, bigender, nongender, agender, polygender) [2]. The issue of sex and gender definition in medical research is further complicated by gender transition and its meanings, which may vary from social transition (e.g., change of appearance, pronoun, and name), medical transition (e.g., hormonal treatments), or surgical transition.

It has been recently underlined that integrating sex and gender analysis into the design of research can improve research methodology. Particularly, Tannenbaum et al. [1] extensively discussed how, in different research fields, disaggregated sex and gender analysis can improve reproducibility through rigorous and specific reporting of data on sex and gender, reduce bias, and offer opportunities for scientific discovery. The proven importance of sex and gender analysis has led many government-led funding agencies, such as the European Commission or the US National Institutes of Health, to change their science policies, asking applicants to explain whether sex and gender analysis is relevant in their research and to justify if not [1].

However, while sex differences are widely investigated in dental research, the influence of gender has not gained enough consideration. Yet, and in most cases, the two terms (gender and sex) are used indiscriminately as synonyms. This issue was also raised in closely allied fields in dental research, such as paleopathology, where the observed differences among males and females in the epidemiology of caries are a representative example of how important it is to consider gender and sex factors separately. Studies on skeletal series showed that, with the onset and intensification of agriculture, women experienced a greater increase in caries prevalence, greater rates of disease progression, and a faster decline in dental health. These probably rely on gender-based division of labor and dietary habits (namely, women's greater involvement in food processing activities, and, therefore, the more frequent access to soft and processed food) but may also be due to hormonal changes (namely, the increase in fertility with sedentism). This sex-based difference is also supported by the greater dental disease burden observed in females after adolescence [3]. As shown, a biocultural perspective is paramount for a deeper understanding of oral diseases in the past, and the same concept can be applied to the present.

In the present narrative review, we try to answer the questions:

- What is the usefulness of considering gender and sex in dental research?
- Which guidelines have been given until now about this topic?
- Do the first 20 scientific dental journals (according to Journal Citation Indicator classification) [4] promote sex and gender analysis in their guidelines?

## 2. Importance of Differentiating Gender and Sex Analysis in Dental Research

To our knowledge, no study investigated the relevance of differentiating gender and sex analysis in dental research. However, we can hypothesize that such a strategy would lead to the above-mentioned advantages in the field of dentistry, too. Moreover, many pathologies, conditions, and risk factors that are relevant in dentistry have a sexual basis, and it is worth considering gender analysis for further studies.

Men and women, for example, exhibit different lifestyle factors such as smoking, which is a proven risk factor for periodontitis. Besides the gender-based differences in the epidemiology of smoking, with a higher prevalence of tobacco use among men due to gender norms, sex/gender differences have also been reported for smoking-related morbidity and mortality, and factors affecting smoking cessation [5]. Female subjects exhibited a higher risk of smoking-related morbidity and mortality, namely regarding the risk of cardiovascular disease (probably due to the function of sex hormones and to the higher smoking-related effects of arterial hypertension and endothelial dysfunction in women), and the risk of specific cancer, such as colorectal neoplasia and bladder cancer [6, 7]. Moreover, women were reported to encounter increased gender- and sex-related barriers to smoking cessation, which include weight gain and the effect of sex hormones (primarily estradiol and progesterone) on smoking addiction and craving. Also, mood and personality can influence men and women differently during the smoking cessation process, and different personality patterns were identified as success predictors in men and women. Therefore, differences in the success of the different types of intervention were reported, with nicotine replacement therapy being more beneficial for men, and non-nicotine medications or behavioral interventions more beneficial for women [5].

Similarly, cardiovascular diseases, which have a bidirectional association with periodontitis, affect more males than females, but females exhibit higher mortality and worse prognosis after acute cardiovascular events. A recent review discussed the possible sex-related causes of this disparity, which is probably due to different cardiovascular physiology [8], but a gender-related and intersectional analysis is needed.

Sex differences in immunity have been extensively reported, and they rely on their genetic and hormone-mediated basis [9]. Dental health is also influenced by the status of the immune system, especially for periodontal diseases where an inflammatory response of the host occurs. Therefore, gender and sex should always be considered as variables in the design of dental research, in order to underline differences, eventually.

Differences in the neuro-immune system may also be on the basis of different pain experiences and the prevalence of chronic pain. Orofacial pain syndromes, such as burning mouth syndrome and temporomandibular disorders are more common among women. Sociocultural components of how pain symptoms are reported (pain), and how physicians understand and treat pain according to patients' gender must also be taken into consideration [10, 11].

Eating behavior, which also shows biological sex differences and gender-based differences, due not only to cultural norms but also to the individual's education, occupational and family function [12], influences oral health: frequent assumption of fermentable carbohydrates leads to unbalanced microbiota and formation of carious lesions while eating disorders, such as bulimia with self-induced vomiting, can heavily affect the integrity of tooth hard tissues, leading to dental erosion and a higher risk of developing carious lesions. The effect of gender-based dietary habits was also highlighted by paleopathology studies, which showed how caries prevalence in women increased across the transition to agriculture [13, 14].

Some developmental anomalies or deformities (e.g., tooth agenesis) which affect the oral apparatus are linked to genetic factors and are reported in the literature to be more prevalent in the female sex, [15] making it fundamental to correctly register the patient sex.

It should also be considered that gender may influence the access to healthcare services, also because of its intersection with other socio-demographic parameters, such as socioeconomic status. For example, the cost of oral healthcare services are more likely to represent a barrier for women than for man, as well as language barriers; however, women are more likely to attend dental care [16]. Transgender and nonbinary individuals may experience greater barriers when accessing healthcare services, due to the fear of discrimination, as reported in a recent survey [17].

### 2.1. How to Report and Integrate Gender/Sex Analysis in Dental Research

In the last few years, the editorial boards of biomedical journals have started to develop and adopt specific policies for sex and gender analysis and reporting. In 2016, The Lancet published a proposal of guidelines for reporting sex and gender in medical journals and included them in their editorial policy [18]. Some other editorials addressing this issue have been published previously [19, 20]. Shortly thereafter, more detailed guidelines were published, the recommendations of the International Committee of Medical Journal Editors (ICMJE) and the Sex and Gender Equity in Research (SAGER) guidelines [21], which were then endorsed by several biomedical journals. In human studies, the main points of such guidelines can be summarized as follows:

- Authors should use the terms sex and gender correctly; the first should be used when reporting biological factors, while the second refers to identity, psychosocial, or cultural factors.
- Sex and gender should always be considered as experimental variables. The study should be designed in a way that can reveal sex-related and gender-related differences, even if they are not initially expected.
- The study population must be inclusive and representative; demographic data of the study population, including sex and gender, should always be reported. The inclusion or reporting of only one sex must be justified, unless the condition investigated affects only one sex (e.g., prostate disease). If the results of the study are to be applied to only one sex or gender, this should be specified in the title or abstract.
- The methods used to obtain information about the sex and gender of the participants should be described (e.g., self-reported, the investigator observed, and laboratory test).
- Data should be routinely reported disaggregated by sex and gender; both sex- and gender-based analyses should be performed, reported, and discussed. In clinical trials, data on withdrawals and dropouts should also be reported disaggregated by sex.
- Null findings should also be reported.

As for incorporating sex and/or gender in experimental design and analysis, Tannenbaum et al. [1] described many aspects of this issue in different disciplines (data reporting, disaggregating data, sample size, sex-/gender-based interactions between participants and between participants and researchers, and so on). In 2020, the European Commission [22] has described in detail how to analyze sex, gender, and their relations in research, including medical research, in an attempt to provide methodological guidelines. In the following paragraph, we try to summarize the current standards for gender-sex analysis, which should be applied to dental research as well.

Sex should always be considered a biological variable. When developing a scientific hypothesis, we should evaluate the biological plausibility of sex emerging as an important variable in the research question and whether it should be considered a covariate, a confounder, or an explanatory variable. The importance of reporting the sex of the research subject is underlined, even in single-sex studies, to allow meta-analysis, identify research gaps, and avoid generalization between sexes. When including subjects, researchers should consider that the prevalence of intersex individuals is quite high in the population, ranging between 1 : 100 and 1 : 4,500 [23, 24]. Thus they should register and report sex as a nonbinary variable. Sex classification should be defined before data collection. It is recommended to report sex-disaggregated data to facilitate future meta-analysis and to provide more valid data to be interpreted.

Gender should also be considered as a variable in all research contexts. When analyzing gender, researchers should first identify which specific dimension of the gender concept (norms, identity, or rules) is more relevant to the specific question asked in the research, to select appropriate instruments of data analysis. Some instruments have been developed lately to analyze more than one dimension [25–27], even though sometimes a single in-depth instrument may be a better choice.

When a correlation exists between sex and/or gender and the research results, it should be investigated if sex and/or gender act as explanatory variables or confounders. Null findings should also be reported in order to reduce publication bias.

As pooling data collected from all sexes and all genders can mask sex differences, it may appear that disaggregating data forces researchers to at least double the number of study participants. Contrariwise, more efficient experimental designs have been described, such as factorial designs which allow only a slight increase in the number of participants (14–33%) [28, 29].

Gender intersects with other social categories like age, socioeconomic status, ethnicity, and reproductive status, and it represents itself as one of the social determinants of health [30]. Thus, it should be evaluated if factors intersecting with sex and/or genders, such as diet, physical activity, use of tobacco, alcohol, or other drugs, education, professional status, and socioeconomic means, might be relevant to the research and need to be analyzed through different designs, which include multiple stratifications and the inclusion of interacting terms to complex factorial designs. Recently, Fava et al. [31] highlighted the importance of collecting multidimensional histories of subjects enrolled in RCTs, including broad psychosocial and demographic characteristics. The following social and behavioral factors should be considered [32]: race or ethnic group; countries where people lived or/and live (past and present); history of the family; education (years); work; economic and social status; marital status; social connection or isolation; stressors (violence, loss of work, death in the family or among friends, etc.); diseases (depression, HIV, and so on); physical activity; tobacco use; alcohol use; use of drugs; and sexual and reproductive history (e.g., perimenopause/menopause, current or previous pregnancies, abortion, maternal, fetal, or neonatal complications).

Finally, it is also important to consider the sex and gender of the investigators, when relevant. Participants of a research may be influenced by the sex or gender of the caregiver, for example, they may report lower experimental pain if the investigator is of the opposite sex/gender [33]. Although this may depend on psychosocial reasons in humans, it must be taken into consideration that such phenomena can have a biological basis: for example, in the “male observer effect,” male, and even more in female mice, did not exhibit pain when a male observer was present, due to male-associated olfactory stimuli [34].

On the other hand, the sex/gender of the investigator can also produce biases: for example, female care providers were reported to recommend more psycho-social treatments for pain in women than in men [35]. For these reasons, the research team should be comprised of both women and men, who should be trained to avoid investigator sex-gender bias [33].

Interestingly, a recent review showed a sex-gender difference in placebo and nocebo responses. In particular, men and women respond more strongly to placebo and nocebo, respectively, and their responses are mediated by different stimuli, namely verbal information for men, and conditioning procedures in women [36].

### 2.2. Gender and Sex in Dental Journals

The instructions for authors of the first 20 dental journals with the highest impact factor of 2021 (Clavirate Analytics JCR) were screened to examine if they include guidelines for sex and gender analysis and reporting. If no instruction was present, or if the instructions referred directly to the policy of the publisher, the latter were also screened for specific sex/gender guidelines. The results are reported in Table 1. We found that out of 20 journals only five mentioned both the terms “sex” and “gender” in the authors' guidelines and out of 15 that did not mention them, three referred to the publisher's policy. Out of seven publishers only two have mentioned them in the editorial policy. All the instructions and editorial guidelines, however, specify only that the terms “sex” and “gender” should be used correctly. None of them require or suggest performing a sex and gender analysis of data.

## 3. Conclusion

Gender and sex analysis and reporting is still an issue in medical search in general, as the terms “sex” and “gender” are frequently used as synonyms, and disaggregated data are not reported, making it difficult to reproduce and generalize the study results. Attempts have been made by government-led funding agencies, government bodies, and editorial boards of medical journals to define guidelines for researchers in different fields. As for dentistry, although most dental journals recommend the correct use of the terms sex and gender, and to include a representative population in the experimental setting, specific and differentiated sex and gender analysis and reporting are still not required. The integration of sex and gender analysis in dental research, which is still not routinely applied, may be improved in the future by updating editorial guidelines and developing more specific methodological recommendations.

## Figures and Tables

**Table 1:** Journal and publisher policy regarding sex and gender in the 20 journals with the highest impact factor in dentistry.

Journal	Publisher	Impact factor 2021	Journal guidelines	Editor guidelines
Journal of clinical periodontology	Wiley	8,728	None	None
Periodontology 2000	Wiley	7,589	None	None
Journal of periodontology	Wiley	6,993	None	None
Journal of dental research	Sage publication inc	6,116	None	Correct use of the term gender and sex required and have to be reported
Clinical oral implant research	Wiley	5,977	None	None
Journal of oral microbiology	Taylor and francis	5,474	None	None
Oral oncology	Elsevier	5,337	The term sex and gender should be used correctly, and the use of an inclusive language is recommended	Correct use of the term gender and sex required
Dental materials	Elsevier	5,304	The term sex and gender should be used correctly	Correct use of the term gender and sex required
International endodontic journal	Wiley	5,264	None	None
Journal of prosthodontic research	Elsevier	4,642	None	Correct use of the term gender and sex required
Journal of periodontal research	Wiley	4,419	None	None
Journal of dentistry	Elsevier	4,379	The term sex and gender should be used correctly	Correct use of the term gender and sex required
Journal of oral pathology medicine	Wiley	4,253	None	None
Journal of endodontics	Elsevier	4,171	The term sex and gender should be used correctly to have a representative human population	Correct use of the term gender and sex required
Caries research	Karger publisher	4,056	None	None
Clinical implant dentistry and related research	Wiley	3,932	None	None
Journal of prosthetic dentistry	Elsevier	3,426	None	Correct use of the term gender and sex required
European journal of oral implantology	Quintessence	3,123	None	None
European journal of orthodontics	Oxford academic	3,075	None	None
American journal of orthodontics and dentofacial orthopedics	Elsevier	2,650	The term sex and gender should be used correctly to have a representative human population	Correct use of the term gender and sex required

## Data Availability

The data used to support the findings of this study are included within the article.
